# Retroperitoneal fibrosis in presence of autoimmune coagulation factor XIII deficiency result in recurrent critical post-operative hemorrhage: a case report and molecular research with new insights

**DOI:** 10.3389/fimmu.2025.1591847

**Published:** 2025-07-10

**Authors:** Tomonori Matsushita, Mitsuhiro Tachibana, Hiromichi Nakagawa, Shuhei Goto, Koji Nishizawa, Takashi Kobayashi, Shigeki Fukuzawa, Kunihiko Itoh

**Affiliations:** ^1^ Department of Urology, Shimada General Medical Center, Shimada, Shizuoka, Japan; ^2^ Department of Urology, Kyoto University Hospital, Kyoto, Kyoto, Japan; ^3^ Department of Diagnostic Pathology, Shimada General Medical Center, Shimada, Shizuoka, Japan; ^4^ Department of Urology, Kyoto University Graduate School of Medicine, Kyoto, Kyoto, Japan; ^5^ Department of Clinical Pharmacology & Genetics, School of Pharmaceutical Sciences, University of Shizuoka, Shizuoka, Shizuoka, Japan

**Keywords:** autoimmune coagulation factor XIII deficiency, retroperitoneal fibrosis, plasma exchange, IgG4-autoimmune disease, IgG4-related disease, post-operative recurrent bleeding, case report

## Abstract

**Background:**

Idiopathic retroperitoneal fibrosis is an IgG4-related disease where pathological role and clinical significance of IgG4 antibodies remain largely unknown. This report shows a rare case of retroperitoneal fibrosis in presence of Autoimmune coagulation factor XIII deficiency, in which an acute life-threatening hemorrhage was successfully managed with effective treatment strategies. Following this, we investigated the molecular mechanisms underlying the patient’s pathology using experimental translational approach.

**Case presentation and research results:**

The patient was a 60-year-old Asian man with a retroperitoneal mass. A laparoscopic biopsy confirmed that the lesion was retroperitoneal fibrosis with IgG4-expressing plasma cell infiltration. Though biopsy was completed without complications, the patient experienced repeated life-threatening intraperitoneal bleeding starting the next day. Despite performing one emergency laparotomy and three series of emergency transcatheter arterial embolization along with massive transfusions, achieving hemostasis was difficult. Suspecting a humoral autoimmune hemorrhagic disorder, we performed plasma exchange, which achieved complete hemostasis. Later, an abnormal decrease in the activity of coagulation Factor XIII was observed, leading to the diagnosis of Autoimmune coagulation Factor XIII deficiency. Subsequent treatment with steroids and coagulation Factor XIII concentrates prevented further bleeding. We investigated the potential involvement of IgG4-related disease and the effects of IgG4 on coagulation Factor XIII using an *in vitro* system, and it was demonstrated that both IgG1 and IgG4 recognized the A subunit of coagulation Factor XIII. The purified IgG antibody samples containing IgG1 and IgG4 were shown to significantly reduce the function of coagulation Factor XIII derived from healthy individuals.

**Conclusions:**

The patient experienced recurrent life-threatening bleeding due to Autoimmune coagulation Factor XIII deficiency, which was successfully controlled through plasma exchange therapy. This is the first reported case of concurrent retroperitoneal fibrosis and Autoimmune coagulation Factor XIII deficiency. Based on the results of our research, it is suggested IgG4 may play a role in the pathology of both disorders. It was hypothesized that this hematological disorder could be a part of the spectrum of IgG4 autoimmune diseases.

## 1 Introduction

Autoimmune diseases encompass a wide spectrum, including two extensively studied groups characterized by Immunoglobulin G4 (IgG4): IgG4 autoimmune disease (IgG4-AD) and IgG4-related disease (IgG4-RD). These two categories have been clearly distinguished (1). IgG4-ADs features pathological IgG4 antibodies which recognizes specific self-antigens and play a central role in disease progression (2). In contrast, IgG4-RDs are characterized by tumor-like lesions forming in the salivary glands, pancreas, periaortic tissues, or other organs. These lesions are typically infiltrated by IgG4-positive plasma cells with elevated serum IgG4 levels (3–6), though the antigens recognized by these IgG4 antibodies remain unclear. While the immunological pathology is uncertain, these diseases often present challenges in differentiation from other tumor-like lesions and can cause organ dysfunction due to mass effects. One example of IgG4-RDs is idiopathic retroperitoneal fibrosis (RPF), which can lead to non-immunological conditions such as ureteral obstruction (7). Due to its anatomical location and tendency to involve the urinary tract, RPF is often diagnosed and treated in the field of urology. While specific guidelines may vary, a biopsy is generally required for establishing the definitive diagnosis of IgG4-RD (5, 6). Based on the location of RPF, a CT-guided or laparoscopic biopsy is considered to perform for obtaining tissues. When fibrous tissue and infiltration of IgG4-producing plasma cells are observed in the biopsy specimen, the diagnosis of RPF as an IgG4-RD is established. Treatment for RPF typically involves corticosteroids to reduce the fibrotic mass, with immunosuppressive agents being used in cases resistant to steroids. Ureteral stenting or surgical resection may also be performed as adjunctive treatments for ureteral strictures (8, 9).

We suspected RPF in a patient and performed a biopsy to confirm the diagnosis. This intervention led to a clinically severe condition, which we addressed by implementing a course of therapy, and demonstrated its effectiveness. In addition to the clinical results confirming immunological abnormalities played a role in triggering the potentially fatal condition, we investigated the underlying causes of these abnormalities through a molecular research approach.

## 2 Case presentation

**Figure 1:**
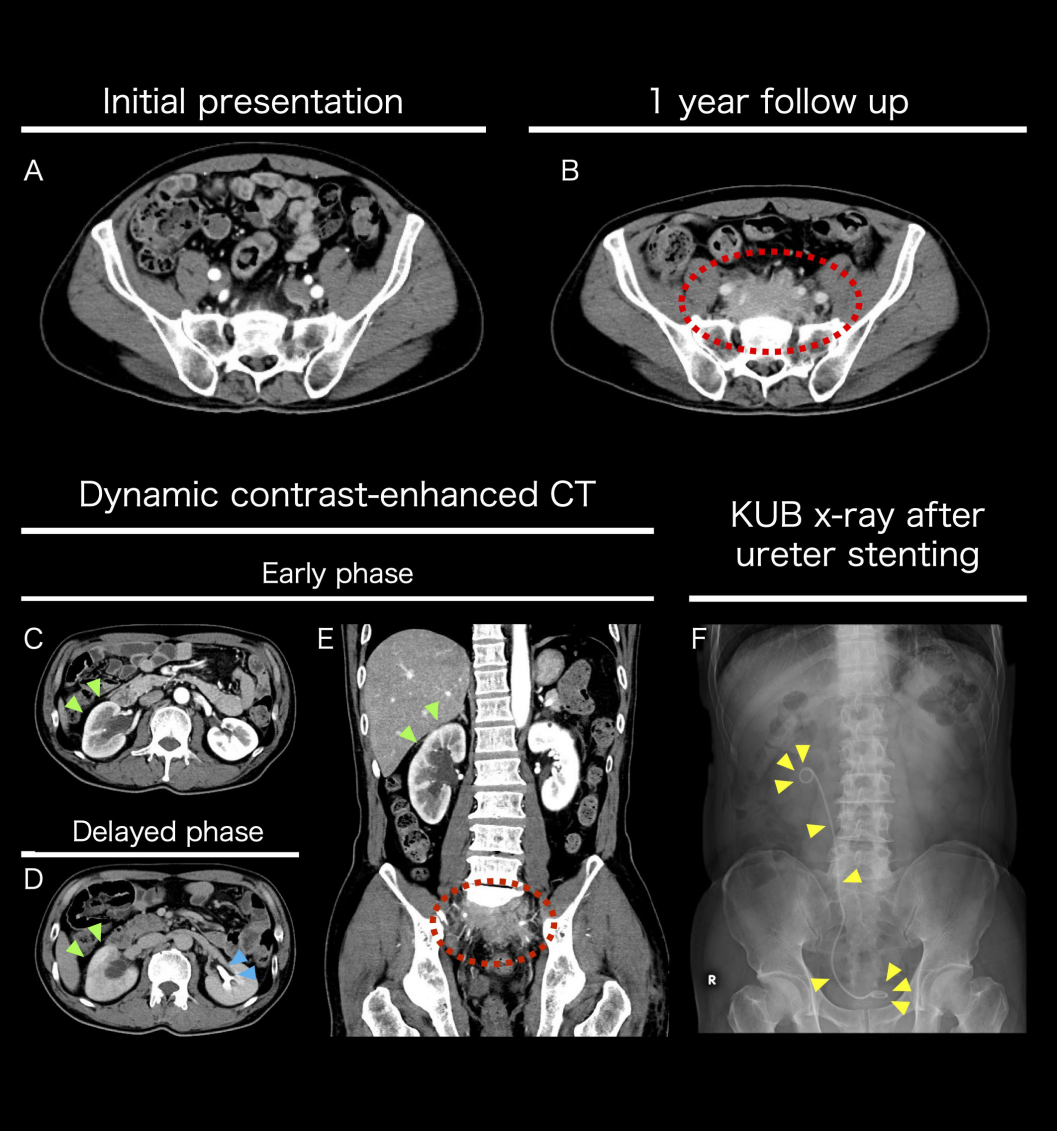
Imaging studies performed pre-hospital admission **(A-F)** Axial contrast-enhanced CT images at the sacral S1 level. Comparative images of the patient at the initial visit **(A)** and after one year **(B)**. A growing mass was observed anterior to the sacrum. Axial dynamic contrast-enhanced CT images at the renal hilum (L2 level). Comparison between the early phase **(C)** and the delayed phase **(D)**. The yellow-green arrow indicates poor contrast enhancement of the right renal cortex, suggesting reduced urine production. The light blue arrow indicates normal function of the left kidney, with urine being produced in the renal pelvis during the delayed phase. Coronal dynamic contrast-enhanced CT image in the early phase **(E)**. The yellow-green arrow indicates reduced contrast enhancement of the right renal cortex, while the dashed red circle marks the mass. The mass caused right ureteral stenosis, leading to hydronephrosis. To preserve renal function, a right ureteral stent was placed transurethrally. Post-stenting KUB X-ray image **(F)**.

A 60-year-old Asian man presented to a local hospital with asymptomatic gross hematuria. His medical history included type II diabetes mellitus, dyslipidemia, gastric submucosal tumor, and clinically insignificant idiopathic cerebral microbleeds. The prescribed oral medications were metformin, anagliptin, and pitavastatin. Physical examination at the initial presentation was unremarkable. Urinalysis confirmed hematuria, but detailed evaluation revealed no obvious causes. Follow-up urinalysis showed spontaneous resolution of hematuria, leading to a one-year follow-up. One year later, a follow-up computed tomography (CT) scan revealed a growing retroperitoneal mass (**Figures 1A, B**). During this period, he experienced weight loss and four episodes of spontaneously resolving subcutaneous hemorrhages. Evaluations by hematological specialist revealed no abnormalities, leaving the cause undetermined.

The retroperitoneal mass caused right ureteral obstruction, necessitating the placement of a right ureteral stent to preserve kidney function (**Figures 1C–F**). An 18F-fluorodeoxyglucose positron emission tomography CT showed uptake in the mass but no significant uptake elsewhere (**Supplementary Figure S1**). Soluble Interleukin 2 receptor and IgG4 blood levels were elevated (641 U/mL and 246 mg/dL respectively). Based on the results of clinical examinations and tests, RPF was the most likely, but other malignant diseases could not be ruled out. As the location was challenging for a CT-guided biopsy, a laparoscopic retroperitoneal mass biopsy was planned.

**Day1-2:** The patient was admitted to our hospital, and the following day evening we performed biopsy successfully without obvious complications (**Supplementary Movie**).

**Figure 2:**
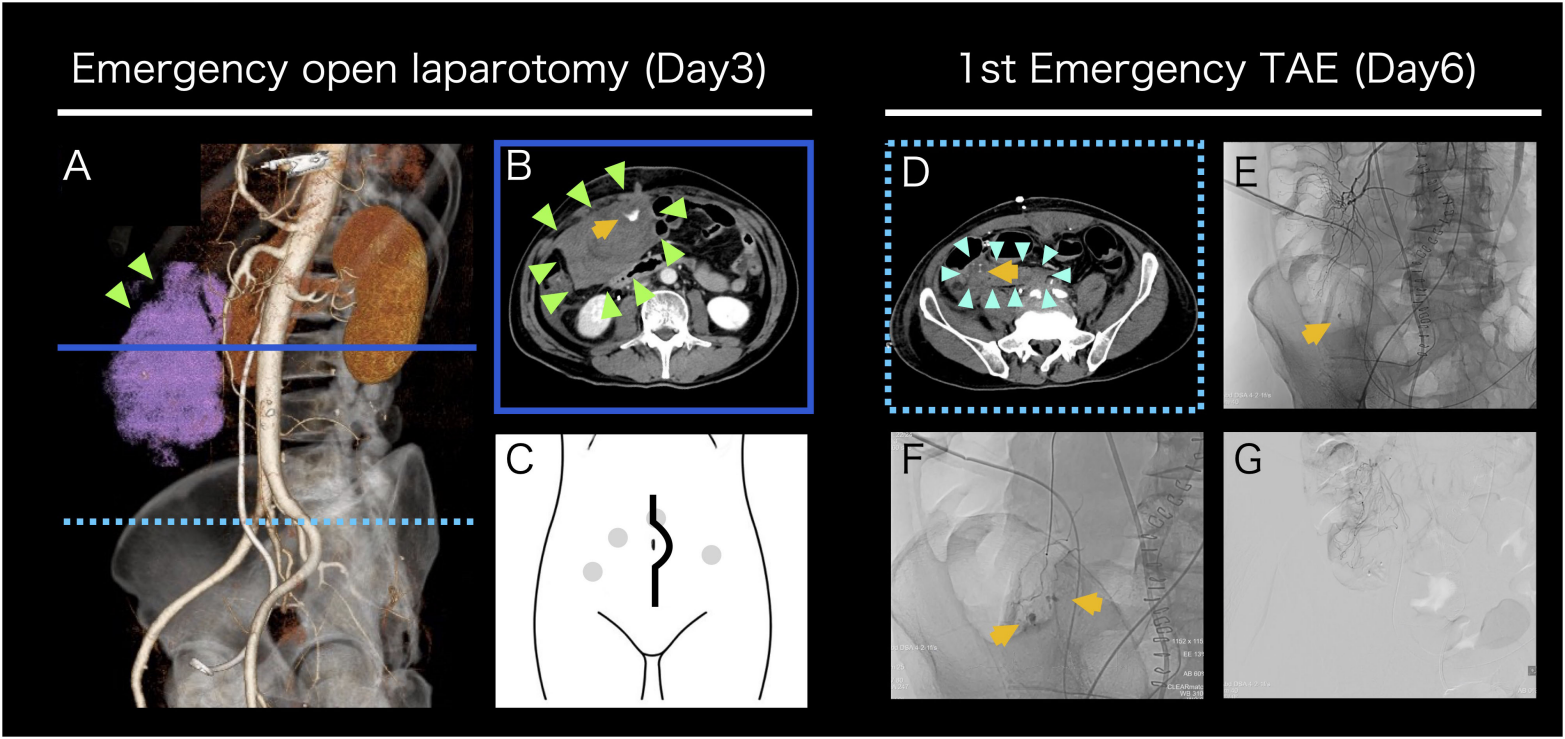
Intervention for post-biopsy bleeding **(A-G)** Contrast-enhanced CT images before emergency open laparotomy **(A, B)**. 3D reconstruction oblique image shows an intra-abdominal hematoma on the ventral side of the right kidney, indicated in pink **(A)**. A horizontal cross-sectional image at the level indicated by the solid blue line in **(A)** shows a large hematoma accumulation and extravasation suggestive of arterial bleeding **(B)**. A schematic diagram of a longitudinal abdominal incision. The gray dots indicate the port sites from the retroperitoneal mass biopsy **(C)**. A contrast-enhanced CT image before initial emergency TAE and TAE images **(D-G)**. A horizontal cross-sectional image at the level indicated by the dashed light blue line in 2A shows a new hematoma formation in an area where no hematoma was observed at the time of open laparotomy **(D)**. A low-magnification angiography image. Arterial bleeding was observed at the site consistent with the CT image **(E)**. A high-magnification angiography image. Multiple sites of arterial bleeding were observed near the terminal ileum **(F)**. A post-embolization digital subtraction angiography image confirmed that arterial bleeding was terminated **(G)**. The yellow arrows indicate extravasation, the light green arrows indicate the initial hematoma, and the light blue arrows indicate the second hematoma.

**Day 3-5:** On the morning following the biopsy, the patient experienced transient loss of consciousness and lower abdominal pain upon attempting ambulation, accompanied by hemodynamic instability. Contrast-enhanced CT (ceCT) demonstrated a significant intraperitoneal hematoma with contrast extravasation near the biopsy port, indicating arterial bleeding (**Figures 2A, B**). Emergency laparotomy was performed. Via a longitudinal incision, the peritoneal cavity was entered, and 1,466 mL of hematoma was evacuated (**Figure 2C**). Although no active arterial bleeding was identified, bleeding was presumed to have been controlled by compression in conjunction with massive transfusion of red blood cells (RBC) and fresh frozen plasma (FFP). Hemorrhagic oozing from the posterior peritoneal wall and ileocecal region manipulated during the biopsy, was addressed appropriately. A drain tube was placed via the right lower quadrant, and the procedure was completed. Disseminated intravascular coagulation (DIC) was considered in the differential diagnosis; however, Platelet counts, prothrombin time (PT), activated partial thromboplastin time (APTT), and fibrinogen were within normal limit (287,000/μL, 14.8 seconds, 26.2 seconds, and 347 mg/dL respectively), making DIC unlikely. The patient was admitted to the high care unit (HCU) and remained hemodynamically stable for the following two days.

**Day 6:** On postoperative day 3, a sudden increase in drain output was observed during the early morning hours. The patient developed shock status (Blood Pressure: 82/60 mmHg, Heart Rate 120 bpm), and laboratory evaluation revealed acute anemia. Platelet counts and coagulation parameters were within normal limits. Repeat ceCT identified active arterial bleeding from a different site than previously observed (**Figure 2D**). Emergency transcatheter arterial embolization (TAE) was performed. Angiography revealed multiple bleeding points originating from branches of the superior mesenteric artery, which were selectively embolized using 12.5% N-Butyl-2-Cyanoacrylate (NBCA) -Lipiodol. Super-selective embolization was employed to prevent ischemic complications in the ileum and ascending colon (**Figures 2E–G**). Hemodynamic stability was achieved post-TAE. Given the potential for platelet dysfunction, platelet transfusion was administered. Urine output was 1,010 mL/day, and drain output was 1,610 mL/day.

**Figure 3:**
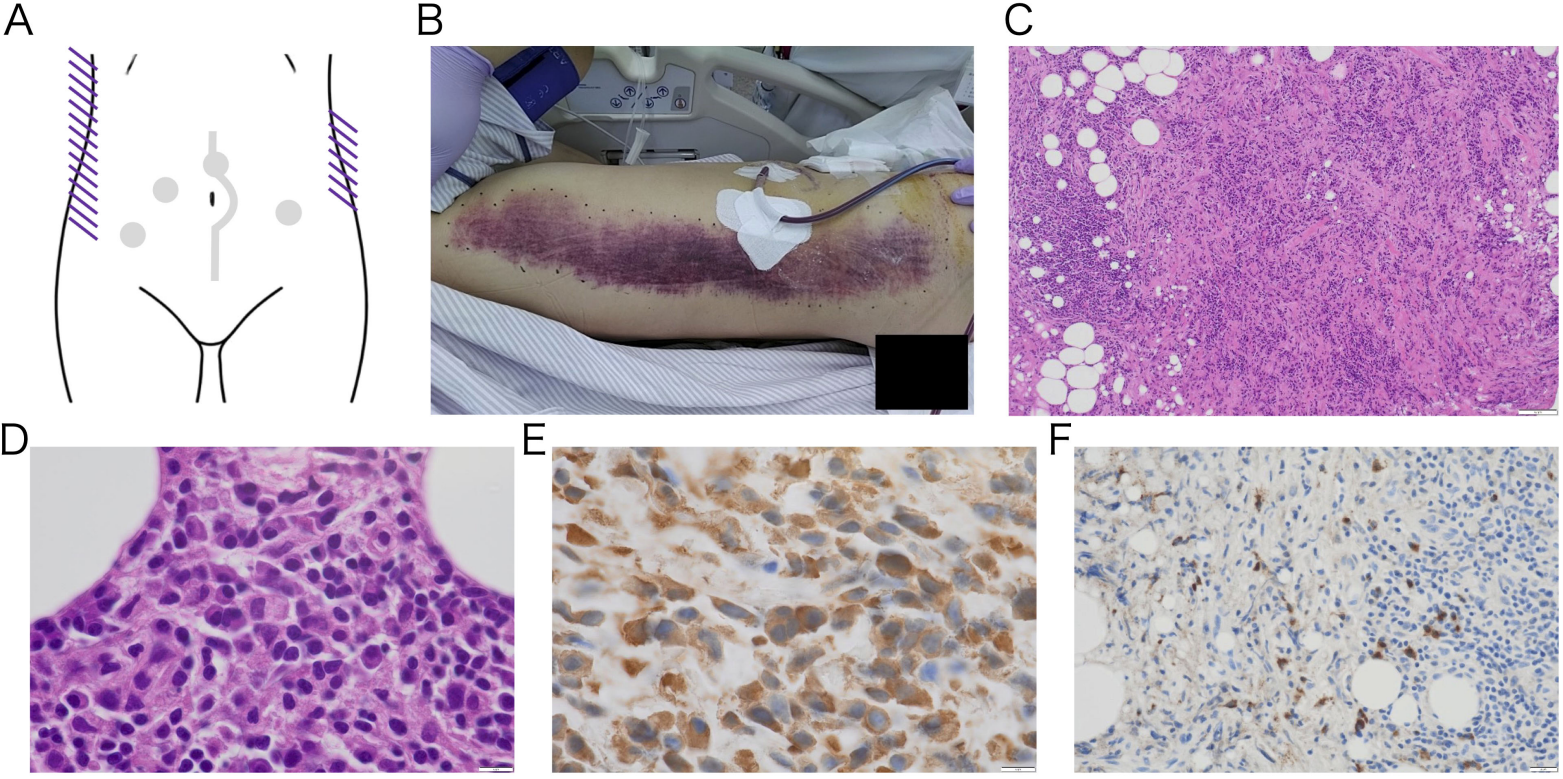
Gross image of subcutaneous hemorrhage on the trunk **(A-B)** and pathological image of retroperitoneal biopsy tissue **(C-F)** Macroscopic image of purpuric lesions that suddenly appeared on both sides of trunk after platelet transfusion. The lesions developed spontaneously, with no signs of trauma. A schematic diagram shows ecchymosis as purple lines. The gray dots and line indicate the previous intervention sites. **(A)** The body image shows right side of his trunk. The left side of the image represents the cranial direction, while the top represents the ventral side **(B)**. No malignant tumor was observed, and the lesion mainly consisted of fibrous tissue with inflammation. Low-magnification H&E-stained image (100×) showing fibrosis and lymphocyte infiltration **(C)**. High-magnification H&E-stained and IgG immunostaining image revealing plasma cell infiltration (1000×) **(D, E)**. IgG4 immunostaining shows IgG4-positive plasma cell infiltration (400×) **(F)**. The IgG4/IgG ratio was 58%, and the number of IgG4-positive plasma cell was 57 per high power field.

**Day 7:** In the early morning, the patient again developed hemodynamic instability (Blood Pressure: 84/62 mmHg, Heart Rate: 112 bpm), necessitating initiation of norepinephrine for blood pressure support. Laboratory tests revealed paradoxical thrombocytopenia and progressive anemia. Notably, extensive ecchymosis appeared on both lateral aspects of his trunk (**Figures 3A, B**), which was not directly involved in prior procedures (laparoscopic biopsy, emergency laparotomy, and prior TAE). Emergent ceCT revealed active bleeding from a new arterial source not previously identified (**Supplementary Figure S2**). The 2nd TAE was performed, revealing relatively strong bleeding from a branch of the right inferior epigastric artery, which was embolized with 20% NBCA-Lipiodol. No rebleeding was observed from previously embolized vessels, confirming technical success. Although platelet dysfunction was not definitively diagnosed, the patient’s response to platelet transfusion raised strong suspicion for an underlying autoimmune hemorrhagic disorder, including thrombotic thrombocytopenic purpura (TTP) in the bleeding phase. At this point, comprehensive laboratory testing for autoimmune hematologic disorders was initiated. Urine output was 1,030 mL/day, and drain output was 2,088 mL/day.

**Day 8:** The following morning, the patient’s general condition deteriorated significantly, with worsening abdominal pain and distension. Vasopressor support was ongoing. Increased bloody drainage and further decline in platelet count were observed. Repeat ceCT revealed yet another arterial bleeding site (**Supplementary Figure S2**). The 3rd emergency TAE was performed, which identified bleeding from a branch of the left inferior epigastric artery. Hemostasis was achieved using multiple microcoils and gelatin sponge. Re-evaluation of all previously embolized vessels showed no evidence of rebleeding. At the same time, histopathological diagnosis of the retroperitoneal mass biopsy was completed. The specimen revealed dense fibrosis with prominent lymphocytic infiltration and IgG4-positive plasma cell infiltration, leading to a diagnosis of RPF (**Figures 3C–F**). This definitive diagnosis of an autoimmune disorder provided the final rationale for initiating plasma exchange (PE) therapy. Collective evidence gathered through the clinical course, combined with the appearance of ecchymosis after platelet transfusion, paradoxical thrombocytopenia, even normal coagulation test results, and results from pathological examinations all point towards some form of humoral autoimmune hemorrhagic disorder. To address a variety of hemorrhagic autoimmune disorders, we planned PE. The clinical situation had become critical, allowing no margin for delay. The 3rd TAE was completed in the morning, and PE was initiated immediately thereafter. FFP was used as the replacement fluid, and 3,000 mL of plasma was exchanged per session. The PE was completed without complications. Following the first session, there was a marked decrease in drain output, and the urine output exceeded the drain. The patient’s abdominal pain improved, and his vital signs remained stable even after discontinuation of vasopressor support. Urine output was 1,380 mL/day, and drain output was 1,189 mL/day.

**Day 9:** PE was repeated under the same conditions. No further episodes of shock occurred after the initiation of PE, and the drain output continued to decline. Although anemia was still present, RBC transfusion resulted in a reactive increase in hemoglobin levels. The patient’s general appearance improved significantly, and there was no need to resume vasopressor support. Urine output was 2,070 mL/day, and drain output was 838 mL/day.

**Figure 4:**
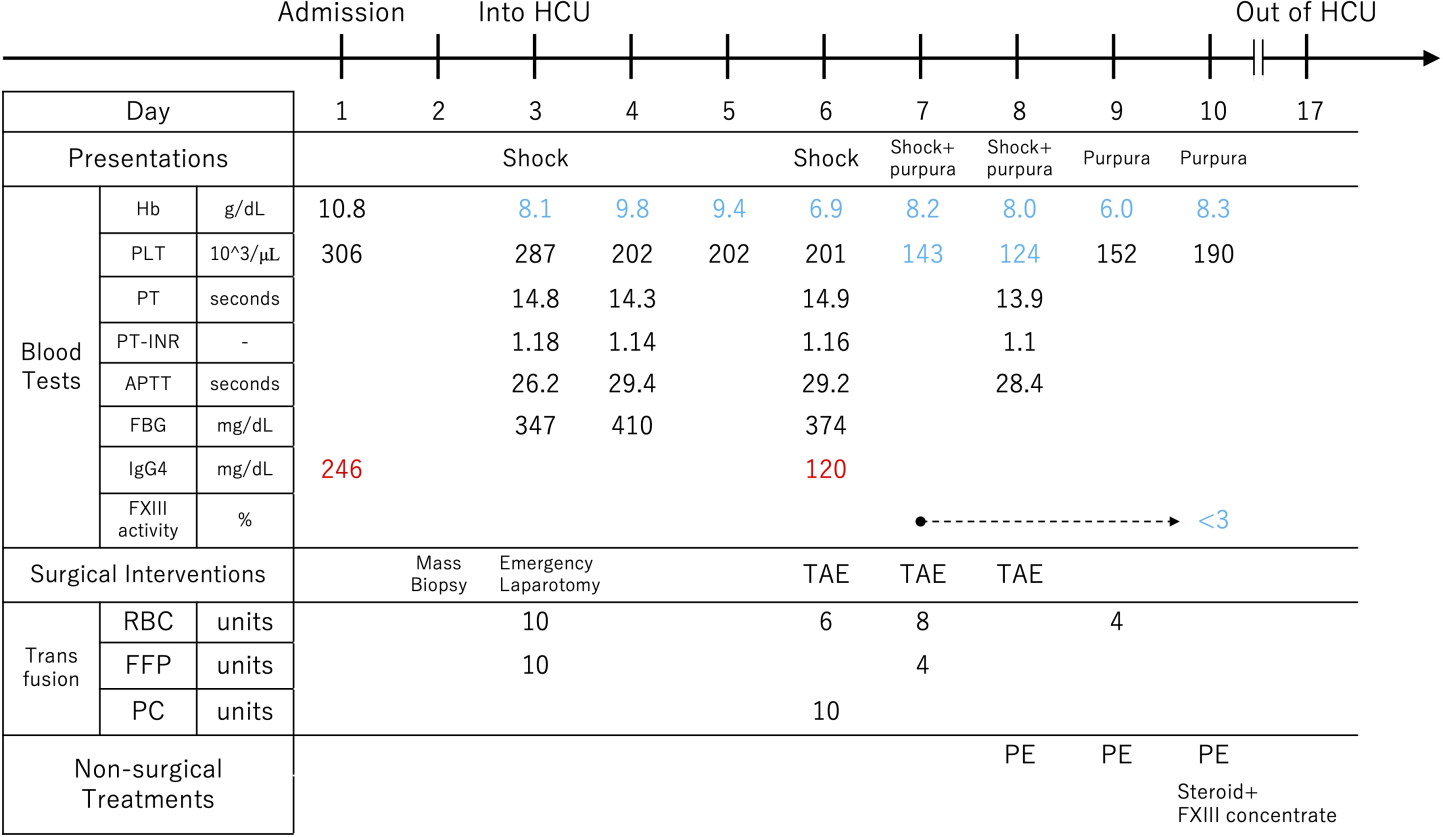
The course from hospitalization to the diagnosis of autoimmune factor XIII deficiency. The factor XIII activity test was submitted on the 7th day of hospitalization, and it was found to be below the detection sensitivity on the 10th day. Hb, hemoglobin; PLT, platelet count; PT, prothrombin time; PT-INR, prothrombin time international normalized ratio; APTT, activated partial thromboplastin time; FBG, fibrinogen; FXIII, coagulation factor XIII; RBC, red blood cell; FFP, fresh frozen plasma; PC, platelet concentrate; TAE, transcatheter arterial embolization; PE, plasma exchange; HCU, High Care Unit; RBC; 140mL/Unit, FFP; 120mL/Unit, PC; 20mL/Unit and 3*10^11counts/Unit.

**Day 10:** PE was performed again on the next day, resulting in three consecutive days of treatment. On the same day, partial results of the blood tests submitted on Day 7 were returned. An abnormal decrease in the activity of coagulation Factor XIII (FXIII) was detected in the patient’s plasma (<3%; normal 70-140%) and in mixing test (10%; the patient plasma: normal plasma = 1: 1), leading to a diagnosis of autoimmune FXIII deficiency (AFXIIID). On the ceCT performed on the same day, extravasation had completely resolved (**Supplementary Figure S2**). Based on this diagnosis, we switched the therapy to steroids and FXIII supplementation, which maintained hemostasis. Urine output was 3,220 mL/day, and drain output was 346 mL/day. The clinical course described above is illustrated (**Figure 4**).

In terms of treatment, steroid administration is the first-line therapy for RPF, and mass reduction is reported. In contrast, AFXIIID is typically treated with both steroid therapy and administration of FXIII concentrates. Since the treatment approach for AFXIIID encompasses that of RPF, we prioritized treatment for AFXIIID. Specifically, we administered 40 mg of steroid daily along with daily infusions of FXIII concentrate (Fibrogammin P I.V. Injection). The patient was discharged from the HCU on Day 17. The patient was closely monitored via vital signs, drain output, FXIII activity, IgG4 levels, and follow-up CT of the retroperitoneal mass. As drainage decreased and only ascitic fluid remained, the drain tube was removed on Day 20, and he was transferred to the hematology department. While IgG4 levels normalized, FXIII activity remained low, ranging from 5% to 10%. After confirming the absence of recurrent bleeding, FXIII concentrate was discontinued on Day 27. Careful observation continued, and since there was no recurrence of bleeding, only steroid therapy was maintained. A follow-up CT about one month later showed shrinkage of RPF. The ureteral stent was carefully removed without recurrence of bleeding or renal dysfunction. No recurrence of hydronephrosis was noted on repeated follow-up ultrasound examinations. Although no further bleeding episodes occurred, FXIII activity remained persistently low, ranging between 5% and 20%. Finally, he was discharged on Day81.

Later on, other blood test results were returned, and helped rule out possibilities of other blood disorders being also present. The patient fulfilled both the ACR and EULAR classification criteria for IgG4-RD (2019) and the Japanese Comprehensive Diagnostic Criteria for IgG4-RD (2020), leading to a diagnosis of IgG4-RD. We hypothesized that the patient’s IgG4 antibodies in the blood recognize FXIII and are associated with the current clinical manifestations. To test this hypothesis, we conducted *in vitro* experiments to verify the relationship.

## 3 Materials and methods

### 3.1 Sample preparation

With the patient’s informed consent to participate in our study, an additional blood sample was collected on the same day as routine monitoring on Day 27. This day corresponded to 19 days after the initiation of PE and 17 days after the start of steroid and FXIII concentrate therapy. Both steroid and FXIII therapies were ongoing at the time, but this day marked the final administration of the FXIII concentrate. The consent covered the following: use of the patient’s samples for research purposes; use and modification of data obtained from the study, including physical images; and anonymized presentation of these materials at academic conferences or in scientific publications. The plasma was immediately frozen after blood collection and thawed for use in experiments. Blood samples from healthy volunteers were also collected with their consent, and the plasma fractions were frozen for storage. Plasma samples and antibodies were diluted using 1x PBS.

### 3.2 Clustering analysis

Data from previous studies were reorganized (*“Table S2. IgG subclasses and reactivity of anti-FXIII autoantibodies*”, Ichinose et al., 2015). Initially, Case 3 and Case 22 were excluded as no IgG subclasses are detected. The data were transformed as follows: “+” → 2, “±” → 1, and “-” → 0. Clustering was performed on both rows and columns using the average linkage method, and the results were visualized in a heatmap.

### 3.3 Cross-mixing assay

The activity of FXIII was evaluated using the *Berichrom FXIII* assay kit, which employs the ammonia release method. The kit requires 400 µL of sample, and the final volume of all samples was adjusted to 400 µL. Mixed samples were incubated at 37°C for 1 hour before testing, following the manufacturer’s protocol. Dilutions of the samples were performed using 1x PBS or pH-adjusted glycine. 0.1M Glycine-HCl elution buffer (pH 2.5) was neutralized with 1M Tris(hydroxymethyl)aminomethane neutralization buffer (pH 10). The mean and standard error were calculated for both the control groups and test sample groups. Paired one-sided t-tests were used to compare the control and test samples.

### 3.4 Indirect enzyme-linked immunosorbent assay for complex detection

We used Enzyme-Linked Immunosorbent Assay (ELISA) to analyze protein interactions and quantify protein levels. The full-length recombinant human FXIII A subunit was diluted to 2 µg/mL with 1x PBS and dispensed into a 96-well plate at 50 µL per well. The plate was coated overnight at 4°C. The wells were washed, and 100 µL of 1x blocking buffer was added to each well, followed by incubation at 37°C for 1 hour. After discarding the supernatant, 50 µL of serially diluted patient or healthy control plasma was added and incubated at 37°C for 1 hour. Wells were washed and 50 µL of mouse anti-human IgG1, IgG2, or IgG4 monoclonal antibody, diluted to 2 µg/mL in 0.05% Tween-PBS, was added and incubated at 37°C for 1 hour. Wells were washed again, and 50 µL of alkaline phosphatase-conjugated anti-mouse secondary antibody diluted 1:1000 in 0.05% Tween-PBS was added and incubated at 37°C for 1 hour. After the final wash, 50 µL of p-nitrophenyl phosphate substrate was added to each well, and color development was measured at A405 using a microplate reader within 30 minutes. Washing steps consisted of five washes with 100 µL of 0.05% Tween-1x PBS. Each sample was measured in triplicate, and the mean and standard deviation were calculated. Unpaired t-tests were used to evaluate the means of patient and control samples at each dilution series.

### 3.5 Sandwich ELISA for quantitative measurement

IgG was purified from the patient’s plasma using an IgG purification kit based on protein A, following the manufacturer’s protocol. IgG1 and IgG4 levels were quantified in pre-purified plasma, post-purified samples, and supernatants obtained during purification. Mouse anti-human IgG1 monoclonal antibody and mouse anti-human IgG4 monoclonal antibody were diluted 1:1000 in 1x PBS and dispensed into a 96-well plate at 50 µL per well. The plate was coated overnight at 4°C. After washing, 100 µL of 1% blocking buffer was added to each well and incubated at 37°C for 1 hour. The wells were then washed, and 50 µL of the sample, diluted 1:1000, was added and incubated at 37°C for 1 hour. After washing, 50 µL of alkaline phosphatase-conjugated rabbit anti-human IgG antibody, diluted 1:1000 in 0.05% Tween-PBS, was added and incubated at 37°C for 1 hour. Wells were washed, and 50 µL of p-nitrophenyl phosphate substrate was added and incubated at 37°C. The absorbance at A405 was measured using a microplate reader. Washing steps consisted of five washes with 100 µL of 0.05% Tween-1x PBS. Each sample was measured in six wells, with the maximum and minimum values excluded. From the remaining four values, the mean of the negative control was subtracted, and the mean and standard deviation of the corrected data were calculated.

1. Mouse anti-Human IgG1 Fc Monoclonal Antibody (clone HP6001), MAB1307, Merck KGaA, Darmstadt, Germany
2. Mouse anti-Human IgG2 Fc Monoclonal Antibody (clone HP6002), MAB1308, Merck KGaA, Darmstadt, Germany
3. Mouse anti-Human IgG4 Fc Monoclonal Antibody (clone HP6023), MAB1312, Merck KGaA, Darmstadt, Germany
4. Alkaline Phosphatase-conjugated Affinity Rabbit Anti-Mouse IgG, Fc**γ** Fragment specific, 315-055-008, Jackson Immuno-Research, PA, USA
5. Human Coagulation Factor XIII A/F13A Protein (HEK293, His), HY-P70266, MedChemExpress, NJ, USA
6. Alkaline Phosphatase-conjugated Affinity Rabbit Anti-Human IgG, F(ab’)2 Fragment specific, 309-055-006, Jackson Immuno-Research, PA, USA
7. SIGMAFAST p-Nitrophenyl phosphate Tablets, N1891, Merck KGaA, Darmstadt, Germany
8. Block Ace, UK-B80, KAC, Hyogo, Japan
9. Ab-Rapid SPiN EX, P-014-10, Protenova, Kagawa, Japan
10. Buffer Kit (pH2.5), P-011, Protenova, Kagawa, Japan
11. Berichrom^®^ FXIII, SIEMENS Healthineers, Marburg, Germany

## 4 Research design and results

**Figure 5:**
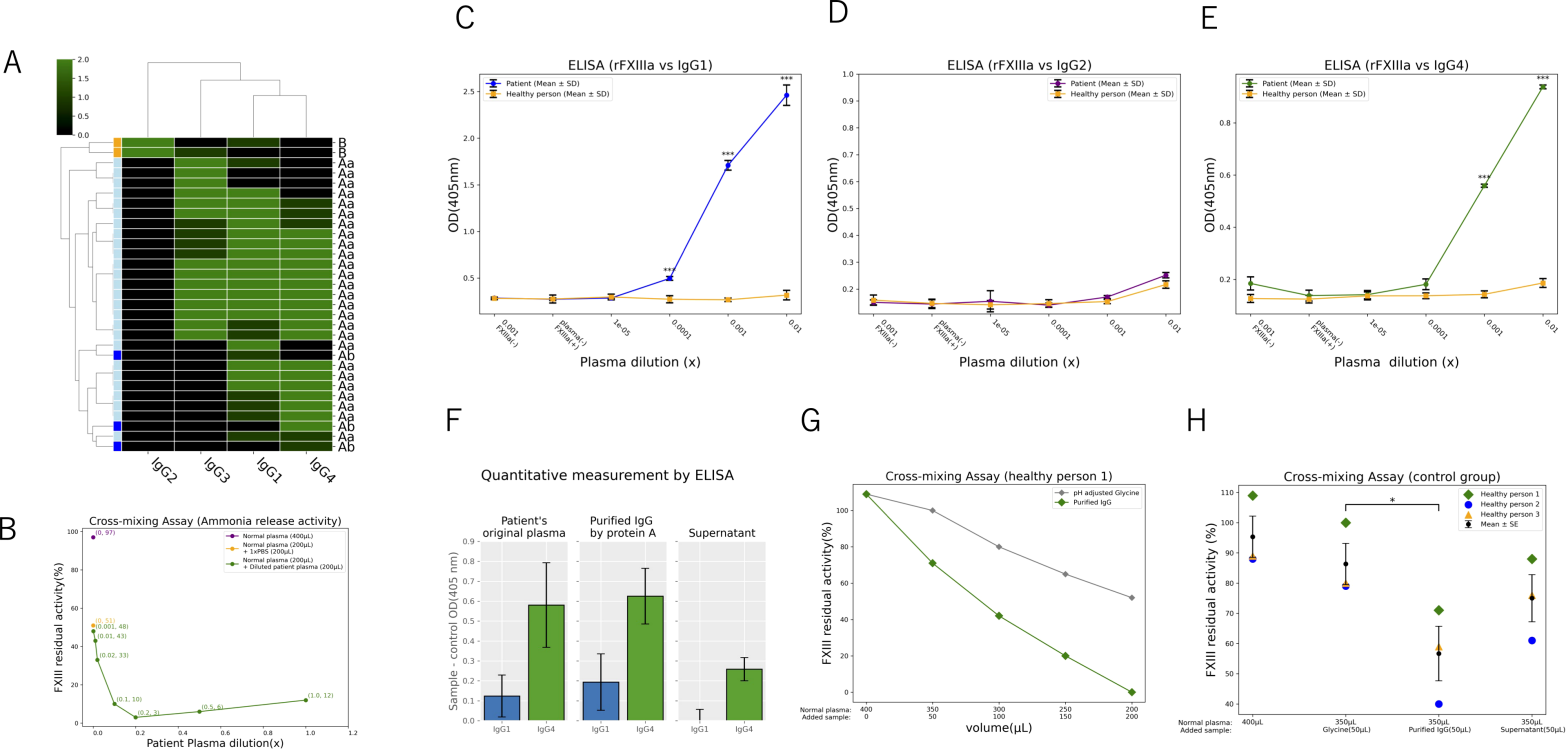
Molecular biological evaluation of the patient’s IgG1 and IgG4 against FXIII **(A-H)** Reorganization of 31 patients’ IgG subclasses and FXIII autoantibodies using hierarchical clustering, visualized as a green-scale heat map **(A)**. Activity curve after mixing normal plasma with serially diluted patient plasma **(B)**. Indirect ELISA detecting complexes between full-length recombinant human FXIII A subunit protein and patient plasma-derived IgG antibodies **(C-E)**. **(C)** shows the evaluation of IgG1, and **(E)** represents IgG4 (***p < 0.01). There is no significance on IgG2 **(D)**. Sandwich ELISA for quantification of IgG subclasses after protein A purification of patient plasma **(F)**. Activity assessment after mixing protein A-purified patient samples with normal plasma, demonstrating a dose-dependent activity reduction **(G)**. Activity evaluation after mixing protein A-purified patient samples with multiple normal plasma samples (*p < 0.05) **(H)**.

There are few papers that mention antibodies against AFXIIID, and only the involvement of IgG1 has been indicated (10). Recently, however, some researchers conducted a detailed study on the functionality and subclasses of antibodies in AFXIIID. The authors classified anti-FXIII autoantibodies into three types - Aa, Ab, and B - based on their recognized subunit and effects. They also investigated the IgG subclasses detected for each type (11). Using these findings, we reorganized them and analyzed the relationship between antibody types and subclasses through hierarchical clustering. As a result of clustering analysis, Type A was strongly clustered with IgG1, IgG3, and IgG4, while Type B formed a cluster with IgG2. Notably, IgG2 was not detected in Type A, and IgG4 was not detected in Type B (**Figure 5A**). The authors also demonstrated in the same study that the type of anti-FXIII autoantibodies could be categorized based on the results from functional assays. At our institution, FXIII activity is evaluated using the ammonia release method. To find out which FXIII subunit the autoantibodies in this patient’s plasma recognize, we performed a cross-mixing assay using a dilution series. The FXIII activity in 400 µL of normal plasma derived from a healthy individual was 97%. In a mixture of 200 µL normal plasma and 200 µL PBS, the activity was reduced to approximately half (51%). Subsequently, the activity was measured in mixtures of 200 µL normal plasma with 200 µL patient plasma. When mixed with patient plasma, FXIII activity was 12% at x1.0 concentration; it was 6% at x0.5, 3% at x0.2, 10% at x0.1, and 33% at x0.02. At lower concentrations, activity was nearly equivalent to that observed with PBS, with values of 43% at x0.01 and 48% at x0.001 (**Figure 5B**). The resulting activity curve closely resembled that of the reported Aa type, strongly suggesting that the FXIII antibodies in this patient also belong to the Aa type. Based on these findings, we conducted further molecular investigations on the FXIII A subunit. Using indirect ELISA, we evaluated whether antibodies derived from the patient’s plasma form complexes with the recombinant human FXIII A subunit. The results demonstrated that the patient’s IgG1 and IgG4 formed significantly more complexes compared to antibodies of the same subclasses from healthy controls while IgG2 did not show complex formation (**Figures 5C–E**). Next, we purified IgG from the patient’s plasma using a protein A-based IgG purification kit, as protein A is known to bind IgG1, IgG2, and IgG4 (12, 13). The concentrations of IgG1 and IgG4 were quantified by sandwich ELISA in the patient’s plasma (original), purified IgG sample, and supernatant, confirming that the target antibodies were successfully enriched (**Figure 5F**). The purified IgG sample was mixed with plasma from a healthy control, demonstrating a dose-dependent decrease in FXIII activity in the healthy control plasma (**Figure 5G**). Plasma from three healthy donors was mixed with the purified IgG sample from the patient, and FXIII activity was measured. Compared to the control group, the group mixed with patient-purified IgG showed a significant reduction in FXIII activity (**Figure 5H**).

## 5 Discussion and conclusions

In this case, we encountered a patient who developed AFXIIID during the diagnostic process of RPF, a subtype of IgG4-RDs. During repeated episodes of life-threatening bleeding following a biopsy, we faced two major clinical challenges: the difficulty of diagnosis and the difficulty of treatment.

In terms of diagnostic difficulties, AFXIIID itself is extremely rare, and despite its potentially fatal bleeding features, routine blood screening tests did not indicate any abnormalities (14). The mechanism for this will be discussed later. Based on the manifestations of ineffective FFP administration, thrombocytopenia after platelet transfusion, the appearance of ecchymosis at non-traumatic sites following platelet transfusion, and the coexistence of autoimmune RPF, we suspected the coexistence of an autoimmune antibody-mediated hemorrhagic disorder. Although AFXIIID was included in the differential diagnosis, other possible disorders could not be ruled out at that point. Additional differential diagnoses included the bleeding phase of TTP, idiopathic thrombocytopenic purpura, and Evans syndrome. However, blood tests required for a definitive diagnosis took time to process, and the patient’s condition was too critical to afford any delay.

In terms of therapeutic difficulties, it was challenging to achieve hemodynamic stability using conventional surgical intervention methods. PE has been shown to be effective for TTP (15), but there are few reports demonstrating its effectiveness in other autoimmune hemorrhagic disorders. In the absence of a definitive diagnosis, we were forced to perform “empiric” PE, which successfully stabilized hemodynamics and allowed us to gain time for making a definitive diagnosis. We were able to make a transition from the bleeding phase to the stable phase, during which a definitive diagnosis of the hemorrhagic disorder was established. Immediately after the definitive diagnosis was confirmed, we switched to the combined therapy of FXIII concentrate and steroids, as reported in previous reports (16), and successfully maintained hemostasis.

To our knowledge, this is the first report demonstrating that PE was effective even in the life-threatening bleeding phase of AFXIIID. On top of that, FXIII activity was below the detection threshold, which was considered one of the worst conditions. Though past studies did not recommend PE (15, 16), the fact that hemostasis was achieved through PE under such circumstances is a noteworthy aspect of this report.

As for the original purpose of this hospitalization, the diagnosis of the retroperitoneal mass was confirmed as RPF, a subtype of IgG4-RDs. This report is also the first to document the coexistence of RPF and AFXIIID. Given the extreme rarity of both diseases, it seemed unlikely that they had independently developed simultaneously. Since both conditions involve antibodies, this led us to suspect a shared pathological mechanism, prompting *in vitro* studies.

First, we consider the findings of previous studies. IgG4 is a subclass of IgG antibodies, but it differs significantly from IgG1, IgG2, and IgG3. Unlike the others, IgG4 lacks pro-inflammatory effects and instead plays a primarily anti-inflammatory role. The Fc region of IgG4 lacks binding sites for complement and effector cells, which is considered one of the factors contributing to its anti-inflammatory properties (17). Classical autoimmune diseases such as systemic lupus erythematosus and dermatomyositis involve abnormal hyperactivation of inflammatory immunity. However, in autoimmune diseases where IgG4 plays a central role, the pathological mechanism is a non-inflammatory immune response (17). These diseases have been proposed as a distinct category called IgG4-ADs. Remarkably, IgG4-ADs include TTP, which was our primary suspected condition, as well as myasthenia gravis, pemphigus vulgaris, subtypes of autoimmune encephalitis, and membranous nephropathy (18). In these diseases, the target autoantigen of IgG4 has a physiological function, and the disease occurs due to its neutralization or functional impairment. Furthermore, most of the forementioned IgG4-ADs have been shown to respond well to PE (19–21). This is likely because IgG4 lacks the ability to activate complement or effector cells, making simple removal of pathogenic IgG4 effective in restoring normal physiological function. In cases where activated molecules or effector cells are involved, PE alone may be insufficient for complete removal.

Next, FXIII is a pro-γ-transglutaminase that consists of two enzymatic A subunits and two carrier B subunits as hetero-tetramer (A2B2) in plasma. During proteolytic activation, thrombin cleaves activation peptide in A subunits and ionized calcium releases the B subunits, allowing FXIII to become activated (22, 23). In a non-proteolytic process, FXIII can be activated by calcium ions within the physiological range (24, 25). FXIII is involved in various reactions in the physiological processes, one of which is the coagulation process (14). FXIII stabilizes fibrin polymers in the final step of coagulation cascade. Because it acts after fibrin formation, a decrease in FXIII activity does not affect routine clinical hemostasis tests such as bleeding time, PT, or APTT (26). This was one of the factors contributing to the diagnostic difficulty mentioned earlier. When abnormalities occur in FXIII, they manifest as FXIII deficiency. In addition to bleeding, reported symptoms include delayed wound healing and recurrent miscarriages (27, 28). This disease is classified as either congenital or acquired, with the acquired form further divided into autoimmune and secondary types. The autoimmune type is extremely rare and is known to be mediated primarily by autoantibodies (29, 30). According to the Japanese cohort study, a total of 64 AFXIIID cases were diagnosed during the 12 years from 2010 to 2021. The prognosis is poor with approximately 20% mortality within one year. Unfortunately, 13% of cases are diagnosed postmortem following fatal acute bleeding (16, 31). However, given the possibility that this condition is included among undiagnosed fatal bleeding cases, its incidence and mortality rate may be underestimated. Additionally, considering that trauma in a patient with decreased FXIII activity can trigger disease onset, its true prevalence could be higher than currently assumed, warranting further epidemiological research. The recommended treatment includes the administration of steroids and FXIII concentrate (14–16, 31). PE is reported to be a less effective choice that has a transient effect in reducing inhibitor (15, 16). Though in case where the definitive diagnosis has been established and the patient is relatively stable with his or her accessible bleeding sites, PE may be inappropriate, if a definitive diagnosis has not been made and other autoimmune hemorrhagic disorders cannot be ruled out, or if bleeding control is difficult, PE should be considered.

As mentioned earlier, AFXIIID has been classified into three subtypes based on the subunit targeted by the autoantibody and its function (11). Studies have examined differences in IgG subclass involvement and activity curves based on detection methods. Interestingly, in A-type (Aa and Ab), where autoantibodies bind to the enzymatic A subunit, IgG1, IgG3, and IgG4 have been detected, with a predominant neutralizing effect. These types show that while the decrease in FXIII antigen levels is relatively mild, FXIII activity is significantly reduced. In contrast, in B-type, where autoantibodies bind to the B subunit, IgG2 is primarily detected, with no IgG4 involvement so far. B-type has been associated with hyper-clearance effects, resulting in reduced FXIII activity due to decreased FXIII antigen levels. Although detailed research findings have been presented, previous studies have not discussed the specific role of individual IgG subclasses. Upon reconsideration, the neutralizing effect observed in A-type may be primarily mediated by IgG4, leading to FXIII activity reduction, but since IgG4 lacks clearance capability, the decrease in FXIII antigen itself remains mild. On the other hand, the absence of IgG4 in B-type suggests that since the B subunit is a structural protein with no enzymatic activity, IgG4-mediated neutralization would not result in immunological abnormalities. Immune abnormalities caused by pathogenic IgG4 may be difficult to detect unless they cause clear clinical manifestations.

Next, we consider the A-type AFXIIID in this patient. Based on the similarity in activity curves obtained using the ammonia-release method, the patient’s autoantibody was suspected to be that of the Aa subtype. Using this finding as a basis, we tested for complex formation between the patient’s plasma antibodies and recombinant human FXIII A subunit via indirect ELISA. The results were consistent with the IgG subclass profile of the previously studied A-type patient group. In this study, we were unable to purify IgG4 alone, preventing us from conclusively proving that IgG4 was the sole factor directly inhibiting FXIII. However, we developed a cell-free experimental system using purified IgG from the patient, minimizing the influence of molecules or cells other than IgG. This system demonstrated that the patient-derived IgG significantly reduced FXIII activity in healthy controls. Considering this result, along with the patient’s favorable response to PE, it is highly likely that this patient’s AFXIIID exhibited characteristics of an IgG4-AD. Although we conducted *in vitro* activity assays in this study, there are inherent limitations in replicating the *in vivo* environment. Factors such as *in vivo* concentrations of antibodies and antigens, tissue architecture including blood flow, immune responses beyond IgG, repair mechanisms, metabolic pathways, and tissue-specific localization of FXIII cannot be fully replicated *in vitro*. Future studies should aim to validate whether pathological bleeding can be reproduced *in vivo*.

Finally, we discuss this patient’s RPF. It is classified as an IgG4-RD. Currently, however, IgG4-RDs and IgG4-ADs are considered entirely separate conditions (1, 18, 20). In fact, reports of their concurrent occurrence are extremely rare (32, 33). Unlike IgG4-ADs, much remains unknown about the role of IgG4 in IgG4-RDs. Some studies have suggested that IgG4 in IgG4-RDs may act antagonistically against pathogenic IgG1, potentially functioning as a protective mechanism (34). Understanding the dual role of IgG4 in pathogenesis and protection is crucial for selecting appropriate interventions for IgG4-RDs. IgG4-RDs are known to involve infiltration of IgG4-positive lymphocytes into various tissues and elevation of serum IgG4 levels. Although it remains unclear whether tissue IgG4 and circulating IgG4 are identical or which appears first, it seems reasonable to assume that the infiltrating lymphocytes secrete IgG4 into the bloodstream. In this patient, it is plausible that IgG4 released from lymphocytes within the fibrotic tissue entered circulation, recognized FXIII, and following the invasive biopsy procedure, ultimately caused massive hemorrhage. In our study, we demonstrated that serum IgG1 and IgG4 antibodies recognized FXIII and significantly reduced its activity. However, we were unable to determine whether IgG4 alone was sufficient to suppress FXIII activity. This leads to two possible hypotheses:

i. Both IgG1 and IgG4 contribute to the inhibition of FXIII activity
ii. IgG1 is primarily responsible for the inhibition, while IgG4 provides only partial suppression of IgG1 activity

If the first hypothesis is correct, AFXIIID may be a subtype of IgG4-AD, with RPF potentially serving as the source of pathogenic IgG4. Conversely, if the second hypothesis holds, RPF may act as an anti-inflammatory defense mechanism against pathological IgG1, aligning with the conventional view that IgG4-ADs and IgG4-RDs are distinct disease entities. Further studies will be required to draw the definitive conclusion as to which of the hypotheses is correct.

## Patient’s perspective

I don’t remember much about the treatment itself, but when I later learned about the situation I had been in, I felt truly terrified. However, I am deeply grateful to the medical team, who never gave up, tirelessly searching for a solution and ultimately saving my life. Thanks to them, especially to Dr. Matsushita, I survived and was able to meet my newborn grandchild. I sincerely hope that this discovery will help save the lives of other patients suffering from the same disorder around the world.

## Data Availability

The original contributions presented in the study are included in the article/**Supplementary Material**. Further inquiries can be directed to the corresponding author.
