# Supplementary material for: Retroperitoneal fibrosis in presence of autoimmune coagulation factor XIII deficiency result in recurrent critical post-operative hemorrhage: a case report and molecular research with new insights
Source: Front Immunol. 2025 Jul 10;16:1591847. doi: 10.3389/fimmu.2025.1591847 (PMC12288730; doi:10.3389/fimmu.2025.1591847)
Supplement: Supplementary file 1 [file DataSheet1.docx]

Supplementary Material

Retroperitoneal Fibrosis in presence of Autoimmune Coagulation Factor XIII deficiency result in recurrent critical post-operative hemorrhage: a case report and molecular research with new insights

Tomonori Matsushita, Mitsuhiro Tachibana, Hiromichi Nakagawa, Shuhei Goto, Koji Nishizawa, Takashi Kobayashi, Shigeki Fukuzawa and Kunihiko Itoh

*** Correspondence:** Tomonori Matsushita [sgh.tmatsushita@gmail.com](mailto:sgh.tmatsushita@gmail.com)

#
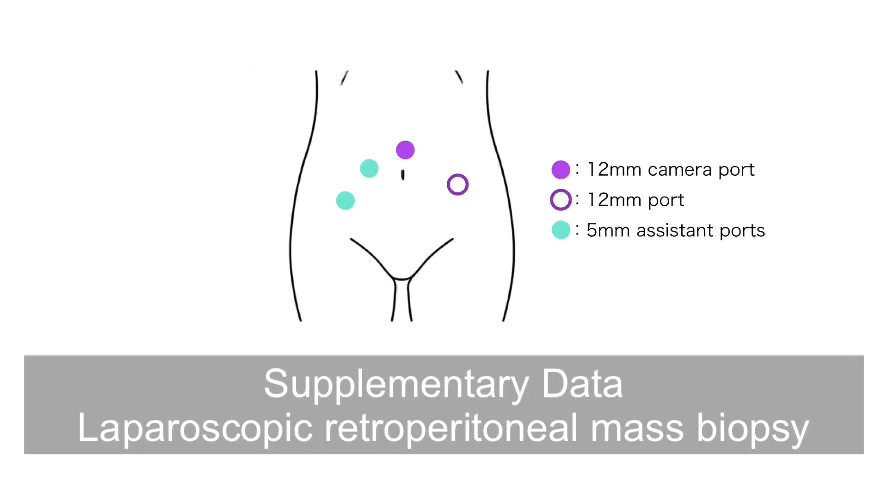
Supplementary Movie

**Supplementary Movie.** Laparoscopic retroperitoneal mass biopsy was performed. Using ultrasound, we confirmed there was no vascular injury. The mass was confirmed to be adequately sampled through rapid pathological diagnosis. The procedure was completed without complications. This movie shows the procedure in detail.

# Supplementary Figure

#

# Figure S1. Whole-body FDG-PET CT showed no significant-uptake lesions other than the retroperitoneal mass identified on the previous CT scan. The left is horizontal, the middle is coronal, and the right is sagittal respectively. The mass is indicated by red color.

# Figure S2. Contrast-enhanced CT follow-up images. The yellow arrows indicate arterial bleeding. The image on the left, taken the day after the initial TAE, showed vigorous bleeding from branches of the right inferior epigastric artery. The middle image, taken the following day, showed bleeding from branches of the left inferior epigastric artery that was not present the previous day. The image on the right is a contrast-enhanced CT scan taken after plasma exchange. While the hematomas from the previous bleeding remained, there was no evidence of new arterial bleeding or extravasation.
